# Diazotrophic abundance and community structure associated with three meadow plants on the Qinghai-Tibet Plateau

**DOI:** 10.3389/fmicb.2023.1292860

**Published:** 2024-01-08

**Authors:** Jean Bosco Nshimiyimana, Kang Zhao, Wenying Wang, Weidong Kong

**Affiliations:** ^1^State Key Laboratory of Tibetan Plateau Earth System, Environment and Resources (TPESER), Institute of Tibetan Plateau Research, Chinese Academy of Sciences, Beijing, China; ^2^College of Resources and Environment, University of Chinese Academy of Sciences, Beijing, China; ^3^Department of Life and Geography Sciences, Qinghai Normal University, Xining, China; ^4^Key Laboratory of Biodiversity Formation Mechanism and Comprehensive Utilization in Qinghai Tibet Plateau, Xining, China

**Keywords:** biological nitrogen fixation, grassland ecosystems, non-leguminous plants, *nifH* gene, diazotrophs, Qinghai-Tibet Plateau

## Abstract

Symbiotic diazotrophs form associations with legumes and substantially fix nitrogen into soils. However, grasslands on the Qinghai-Tibet Plateau are dominated by non-legume plants, such as *Kobresia tibetica*. Herein, we investigated the diazotrophic abundance, composition, and community structure in the soils and roots of three plants, non-legume *K. tibetica* and *Kobresia humilis* and the legume *Oxytropis ochrocephala*, using molecular methods targeting *nifH* gene. Diazotrophs were abundantly observed in both bulk and rhizosphere soils, as well as in roots of all three plants, but their abundance varied with plant type and soil. In both bulk and rhizosphere soils, *K. tibetica* showed the highest diazotroph abundance, whereas *K. humilis* had the lowest. In roots, *O. ochrocephala* and *K. humilis* showed the highest and the lowest diazotroph abundance, respectively. The bulk and rhizosphere soils exhibited similar diazotrophic community structure in both *O. ochrocephala* and *K. tibetica*, but were substantially distinct from the roots in both plants. Interestingly, the root diazotrophic community structures in legume *O. ochrocephala* and non-legume *K. tibetica* were similar. Diazotrophs in bulk and rhizosphere soils were more diverse than those in the roots of three plants. Rhizosphere soils of *K. humilis* were dominated by Actinobacteria, while rhizosphere soils and roots of *K. tibetica* were dominated by Verrumicrobia and Proteobacteria. The *O. ochrocephala* root diazotrophs were dominated by Alphaproteobacteria. These findings indicate that free-living diazotrophs abundantly and diversely occur in grassland soils dominated by non-legume plants, suggesting that these diazotrophs may play important roles in fixing nitrogen into soils on the plateau.

## Introduction

The nitrogen cycle is one of the most important biogeochemical processes on Earth involving microbial activity in the environment. Biological nitrogen fixation contributes 43.2% of nitrogen input in terrestrial ecosystems and is critical to the global nitrogen cycle (Fowler et al., 2013). This process converts atmospheric nitrogen into a form that is biologically accessible to plants. The main source of naturally fixed atmospheric nitrogen, apart from anthropogenic contributions, is biological fixation by diazotrophs, including symbiotic, associated, and free-living nitrogen fixing microbes (Bahulikar et al., 2014; Moyes et al., 2016). Diazotrophs carry the *nifH* gene, encoding the nitrogenase subunit that fixes nitrogen (Chen et al., 2019). The most common symbiotic diazotrophs are Rhizobia, which form symbiotic relationships with legume plants by forming root nodules. They mainly include members of Alphaproteobacteria (such as *Mesorhizobium*, *Rhizobium*, and *Bradyrhizobium*) and Betaproteobacteria (such as members of *Burkholderia* and *Cupriavidus*) (Moulin et al., 2015). Additionally, members of the genus *Frankia* can fix nitrogen by establishing symbiotic associations with nodulated actinorhizal plants (Wall, 2000). Non-symbiotic diazotrophs, which have been found living freely in the soil or associated with plants, may increase nitrogen input for terrestrial ecosystems, particularly those lacking leguminous plants (Rui et al., 2022). In contrast to symbiotic diazotrophs that reside within root nodules (Tu et al., 2016), associative diazotrophs are found on root surface (Bashan et al., 2004), and their associative relationships are characterized by low specificity (Van Dommelen and Vanderleyden, 2007).

Symbiotic and non-symbiotic diazotrophs contribute significantly to fixed nitrogen availability in terrestrial ecosystems. However, despite their lower nitrogen fixation rate, associative diazotrophs are more important than symbiotic diazotrophs globally (Rui et al., 2022). This is because they are more abundant and widespread and have a higher potential to provide most of the fixed nitrogen in different ecosystems, including deserts, tropical forests, and temperate grasslands (Cleveland et al., 1999; Zehr et al., 2003; Gaby and Buckley, 2011). It is estimated that both legume-associated symbiotic diazotrophs and non-symbiotic diazotrophs fix approximately 198 Tg of nitrogen per year on a global scale in natural terrestrial and aquatic environments (Fowler et al., 2013). Therefore, biological nitrogen fixation can alleviate the negative effects of nitrogen limitation in marine and terrestrial ecosystems (DeLuca et al., 2002).

The Tibetan Plateau covers an area of approximately 2.5 × 10^6^ km^2^, making it the highest and largest plateau in the world, two thirds of which are grasslands, including wetlands, meadows, and steppes (Li et al., 2021). Non-leguminous plants such as *K. pygmaea* predominate in the meadow and *Stipa purpurea* in the alpine steppe (Jin et al., 2013). Previous studies have found low nitrogen levels in the Qinghai-Tibet Plateau grassland ecosystems (Shaver and Chapin, 1995; Shaver et al., 2001), however, recent studies have shown that the meadow soils are rich in nitrogen (Guo et al., 2015; Dai et al., 2022). Given that soil nitrogen strongly influences grassland productivity, it is crucial to understand the underlying factors and mechanisms that affect nitrogen levels in an alpine ecosystem.

Legume-associated diazotrophs contribute considerably more to nitrogen fixation than non-symbiotic diazotrophs in various terrestrial ecosystems (Bohlool et al., 1992). However, the legumes account for only 3.4% of the total plant biomass in the Qinghai-Tibet Plateau meadow, due to the low temperature, moisture and limited soil nutrients (Zong et al., 2020). This could limit nitrogen availability for the plants in this area (Jin et al., 2013). Furthermore, studies have shown that the alpine meadows on the Qinghai-Tibet Plateau obtain only 9% of their nitrogen from legumes (Yang et al., 2011). Therefore, the contribution of free-living diazotrophs to nitrogen fixation in this region should not be overlooked.

The availability of nitrogen in Qinghai-Tibet grasslands affects primary productivity similarly to other terrestrial ecosystems (Dong et al., 2016; Mishra and Mainali, 2017). In recent decades, there has been a significant increase in atmospheric nitrogen deposition on the Qinghai-Tibet Plateau (Lü and Tian, 2007; Liu X. et al., 2013), which may help plants overcome nitrogen limitation in this area. However, atmospheric nitrogen deposition is still significantly lower than on other plateaus worldwide (Galloway et al., 2004; Liu Y. et al., 2013). In addition, the Qinghai-Tibet grasslands receive a negligible amount of nitrogen from anthropogenic sources. Therefore, the main source of available nitrogen in the region is likely to be biological nitrogen fixation (Che et al., 2018). Over the years, soil microorganisms in the Qinghai-Tibet Plateau have received much attention due to their crucial role in nitrogen fixation (Deng et al., 2013; Che et al., 2015). Various soil microorganisms, including fungi, bacteria, and archaea, have been well studied in this region (Chu et al., 2016; Shi et al., 2016; Yang et al., 2017). Previous studies have explored the symbiotic diazotrophs associated with legumes on the Qinghai-Tibet Plateau (Che et al., 2018; Zhang et al., 2022; Sun et al., 2023). However, the free-living diazotrophs remain poorly understood. Therefore, this study attempted to determine the abundance and community structure of free-living diazotrophs associated with legume and non-legume plants.

In this study, we investigated the abundance, diversity, and composition of diazotrophs by targeting the *nifH* gene encoding nitrogenase in bulk and rhizosphere soils and roots of the following three plant species: the legume *O. ochrocephala* and the non-legumes *K. tibetica* and *K. humilis*. Our study had the following research questions: (i) whether abundant diazotrophs occur in the rhizosphere and bulk soils of non-legume plants; (ii) are there differences in diazotrophic diversity, composition, and community in legume and non-legume plants; (iii) could non-legumes contribute significantly to nitrogen availability in the Qinghai-Tibet grasslands? Findings from this study would deepen our understanding of the role of free-living diazotrophs in increasing nitrogen input in the Qinghai-Tibet plateau meadow ecosystem. This may also explain why Qinghai-Tibet grassland soils are nitrogen rich despite being dominated by non-leguminous plants.

## Materials and methods

### Study sites and sample collection

Samples were collected at the Haibei Open Research Station of the Chinese Academy of Sciences (37°29′N–37°45′N, 101°12′E–101°23′E) on the northeast Qinghai-Tibet Plateau, a typical alpine meadow ecosystem where *K. humilis* dominates the plant community with *K. tibetica* and *O. ochrocephala*. The average annual precipitation ranged from 426 to 860 mm (Wang et al., 2012). Samples were collected in triplicates for three different plant species. For each plant, three soil cores of 0–10 cm depth and 7 cm diameter were taken from an area of 1 m^2^. The rhizosphere, bulk soil, and roots of each plant were collected in triplicate. The adhering soils were taken as rhizosphere soils after vigorous shaking by hands. Stones were removed from samples using a 2.0 mm sieve, and the plant roots were carefully rinsed under running water to remove adhering soil. Coolers with ice bags were used to transport the samples to the laboratory, where they were kept at −80°C for further analysis.

### DNA extraction and *nif*H gene amplification

The FastDNA® SPIN Kit for Soil (MP Biomedicals, California, USA) was used to extract DNA from 0.3 g of soil or roots by following the manufacturer’s instructions. Prior to DNA extraction, roots were cleaned three times with sterile distilled water, immersed in ethanol for 30 s then rinsed three more times with sterile distilled water. The roots were crushed using sterile silica sand (φ = 0.15 mm) that had been sterilized twice at 120°C for 20 min in a bowl chopper beforehand. DNA purity and concentration were determined using a Nanodrop® ND-2000 UV–Vis spectrophotometer (NanoDrop Technologies, Wilmington, DE).

The *nifH* gene abundance was assessed using the nifH-F/nifH-R primer pair (Rosch et al., 2002) on a Light Cycler 480II thermocycler (Roche, Switzerland). PCR reactions were performed in a 10 μL volume system containing 1 μL of 10x diluted DNA (~10 ng), 0.5 μL of 10 μM each primer, and 5 μL of SYBRGreen Premix (Takara, Japan). The PCR program was set at 95°C for 5 min, followed by 35 cycles of 30 s at 95°C, 30 s at 61°C, and 30 s at 72°C. Fluorescence data were collected at 72°C for 10 s. A standard curve was generated using a serial dilution of plasmids containing the *nif*H gene to calculate the unknown samples as in our previous study (Kong and Nakatsu, 2010). The specificity of the PCR product was checked using a melting curve and agarose gel electrophoresis.

### Terminal restriction fragment-length polymorphism analysis of diazotrophic community structure

The PCR amplifications were performed using the same primer pair described above, with the following conditions: 5 min at 95°C, followed by 35 cycles of 30 s at 95°C, 30 s at 61.5°C, and 30 s at 72°C, and a final extension step of 10 min at 72°C. The reagent 6-carboxyfluorescein (FAM) was used to label the nifH-F forward primer. PCR products were purified by gel electrophoresis using an AxyPrep DNA purification kit (Ax-yGen, USA). After purification, a 30 μL system containing 200 ng of each product and 5 U of *Rsa I* and *Msp I* enzymes was constructed. For enzyme digestion, the samples were incubated at 37°C for 3 h, and the reactions were terminated by incubation at 60°C for 20 min. The Sigma-Aldrich Spin Post Reaction Clean-Up columns (Sigma, USA) were used to purify the digested products, and a mixture of deionized formamide and the internal standard GeneScan-1000 LIZ (Applied Biosystems, USA) was added to a portion of the purified products. The DNA fragments were analyzed using a 3130xl Genetic Analyzer (Applied Biosystems, USA) after denaturing the mixtures at 95°C for 3 min. GeneScan analysis software (Applied Biosystems, USA) was used to determine the Terminal restriction fragment-length polymorphism (T-RFLP) profiles of each sample. Fragment analysis of T-RFLP data was performed between 50 and 460 bp. Before calculating the relative abundance of each fragment, fragments with a peak area of less than 1% were excluded (Lukow et al., 2000). T-RFLP patterns in *K. humilis* roots were used in only one replicate due to the low abundance of *nif*H gene copies detected, as we could not collect sufficient PCR products (200 ng). The OTU sequences obtained from the clone libraries were analyzed *in silico* using the *Msp I* (C^CGG) and *Rsa I* (GT^AC) enzyme sites to determine the phylogenetic affiliation of specific T-RFs.

### Cloning and sequencing of *nif*H gene fragments

A total of four clone libraries for *nifH* gene were constructed to identify the diazotrophs, including two clone libraries from the rhizosphere soil of *K. humilis* and *K. tibetica* and two clone libraries from the root of *O. ochrocephala* and *K. tibetica*. PCR products were amplified using the nifH-F/nifH-R primer set and the same PCR program as described above. The purified PCR products were ligated into the pGEM-T Easy Vector System I (Promega, Madison, WI, USA). The products were transformed into *Escherichia coli* JM109 competent cells by following the manufacturer’s protocol. ABI model 3730xl DNA analyzer (Applied Biosystems, CA) was used to sequence a total of 160 positive clones that were selected randomly from the 4 clone libraries. Multiple sequence alignments were carried out by using CLUSTALW in MEGA6.0 as described by Tamura et al. (2013), and operational taxonomic units (OTUs) were determined based on a 97% sequence similarity threshold using Mothur v.1.33.3 (Schloss et al., 2009). BLASTn^1^ was used to search GenBank for the most closely related sequences to each OTU. The phylogenetic tree was constructed using the neighbor-joining method with a bootstrap with 1,000 iterations in MEGA6.0. The resulting sequences were submitted in the National Center for Biotechnology Information GenBank database under accession numbers KR824430 to KR824503.

### Statistical analysis

Significant difference level of *nif*H gene abundance was measured by one-way ANOVA using SPSS version 19.0 (IBM Inc., USA). All T-RFLP profile fragments were included for principal component analysis (PCA) using CANOCO 5 (Microcomputer Power, Ithaca, NY) to examine the diazotrophic community structure. Shannon diversity was calculated based on T-RFLP profiles using R (version 3.2.2).

## Results

### Diazotrophic gene abundance

**Figure 1:**
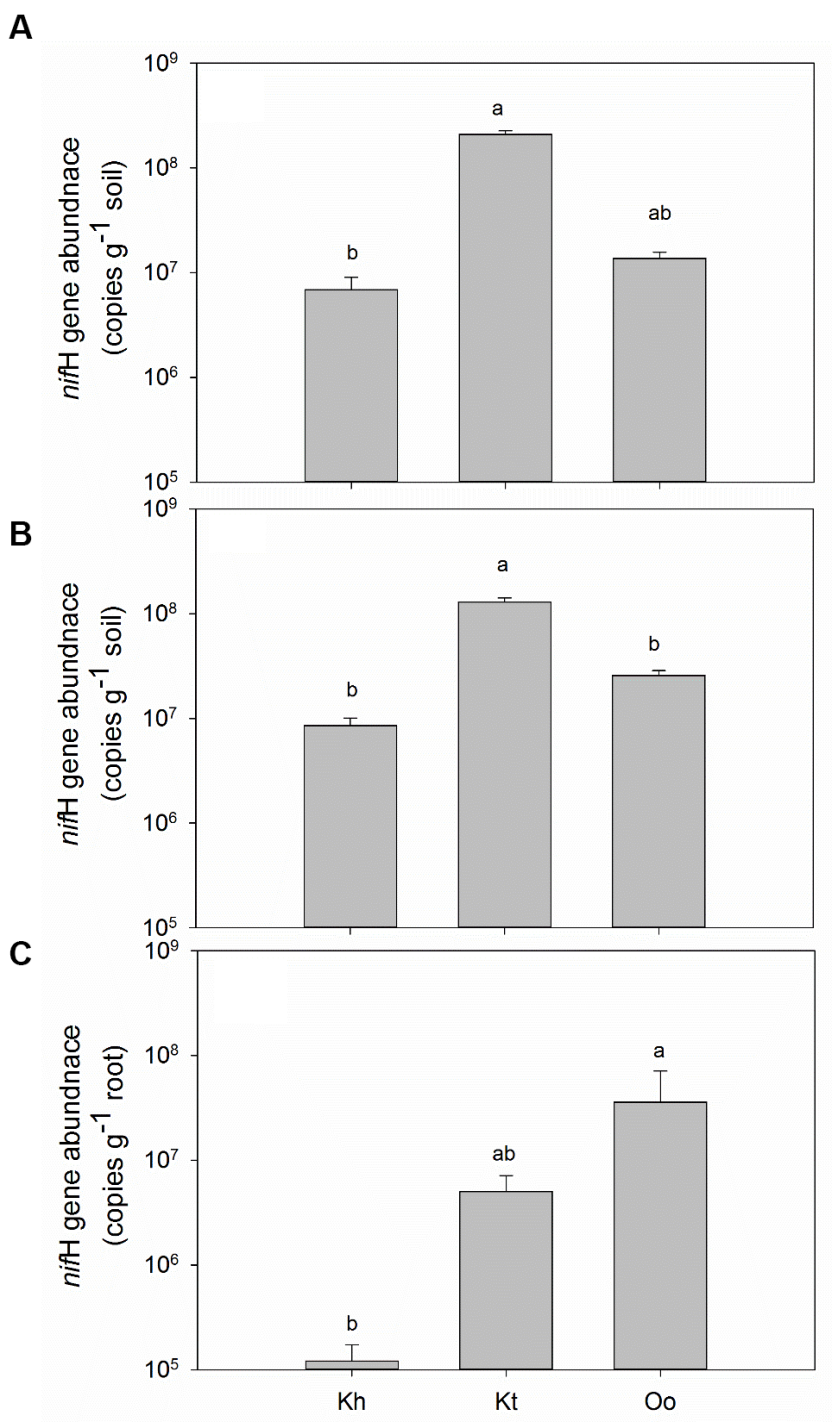
Diazotrophic abundance indicated by the *nifH* gene in the bulk soils **(A)**, rhizosphere soils **(B)**, and the roots of the three plant species **(C)**. Error bars denote the standard error of the mean. Different lowercase letters indicate a significant difference at *p* < 0.05 among the bulk and rhizosphere soils, and roots of three plants. Kh, Kt, and Oo stand for *Kobresia humilis*, *Kobresia tibetica,* and *Oxytropis ochrocephala*, respectively.

The *nif*H gene was detected in all bulk and rhizosphere soils and roots, and the gene abundance was highly dependent on the plant species. In bulk soils, *K. tibetica* showed the highest diazotrophic abundance (2.10 × 10^8^ g^−1^ soil), whereas *K. humilis* showed the lowest (6.84 × 10^6^ g-1 soil, Figure 1A). Similar to the bulk soils, the highest diazotrophic abundance (1.30 × 10^8^ g^−1^soil) was observed in the rhizosphere soils of *K. tibetica*. The lowest was also found in *K. humilis* (8.53 × 10^6^ g^−1^ soil, Figure 1B). In the roots, the legume plant *O. ochrocephala* showed the highest diazotrophic abundance (3.58 × 10^7^ g^−1^ fresh root), which was higher than *K. tibetica* by 7 folds and *K. humilis* by 300 folds (Figure 1C), respectively.

### Diazotrophic community structure and composition

**Figure 2:**
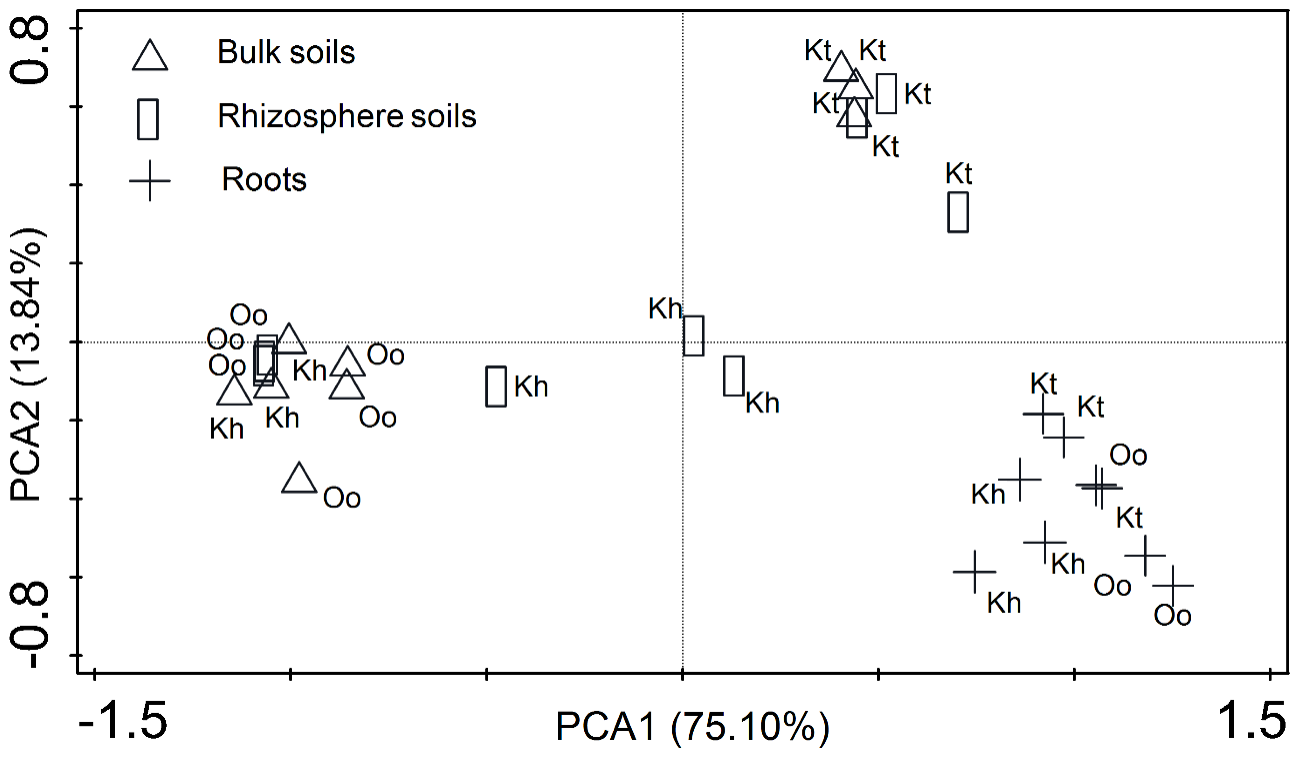
Diazotrophic community structure assessed by principal component analysis (PCA) based on terminal restriction fragment length polymorphism profiles. The shapes of the symbols indicate sample type: bulk soils (triangle), rhizosphere soils (rectangle), and the roots (cross). The percentage of the variation explained by the plotted principal coordinates is indicated on the axes. Kh, Oo, and Kt represent *Kobresia humilis*, *Oxytropis ochrocephala,* and *Kobresia tibetica*, respectively.

Principal Component Analysis (PCA) was used to screen the diazotrophic community structure by including all terminal restriction fragments (TRFs). Both soil and plant types influenced the diazotrophic community structure. In the bulk soil, *O. ochrocephala* had a comparable community structure to the rhizosphere soil, whereas *K. tibetica* had similar diazotrophic community structures in both bulk and rhizosphere soils (Figure 2). The diazotrophic community structure of *K. humilis* was similar to that of *O. ochrocephala* in the bulk soil. In contrast, an independent community structure was found in the rhizosphere soils compared to other samples. The diazotrophic community structures within roots were substantially distinct from the bulk and rhizosphere soils, and the community structures within the roots of the three plant types were similar, independent of plant type.

**Figure 3:**
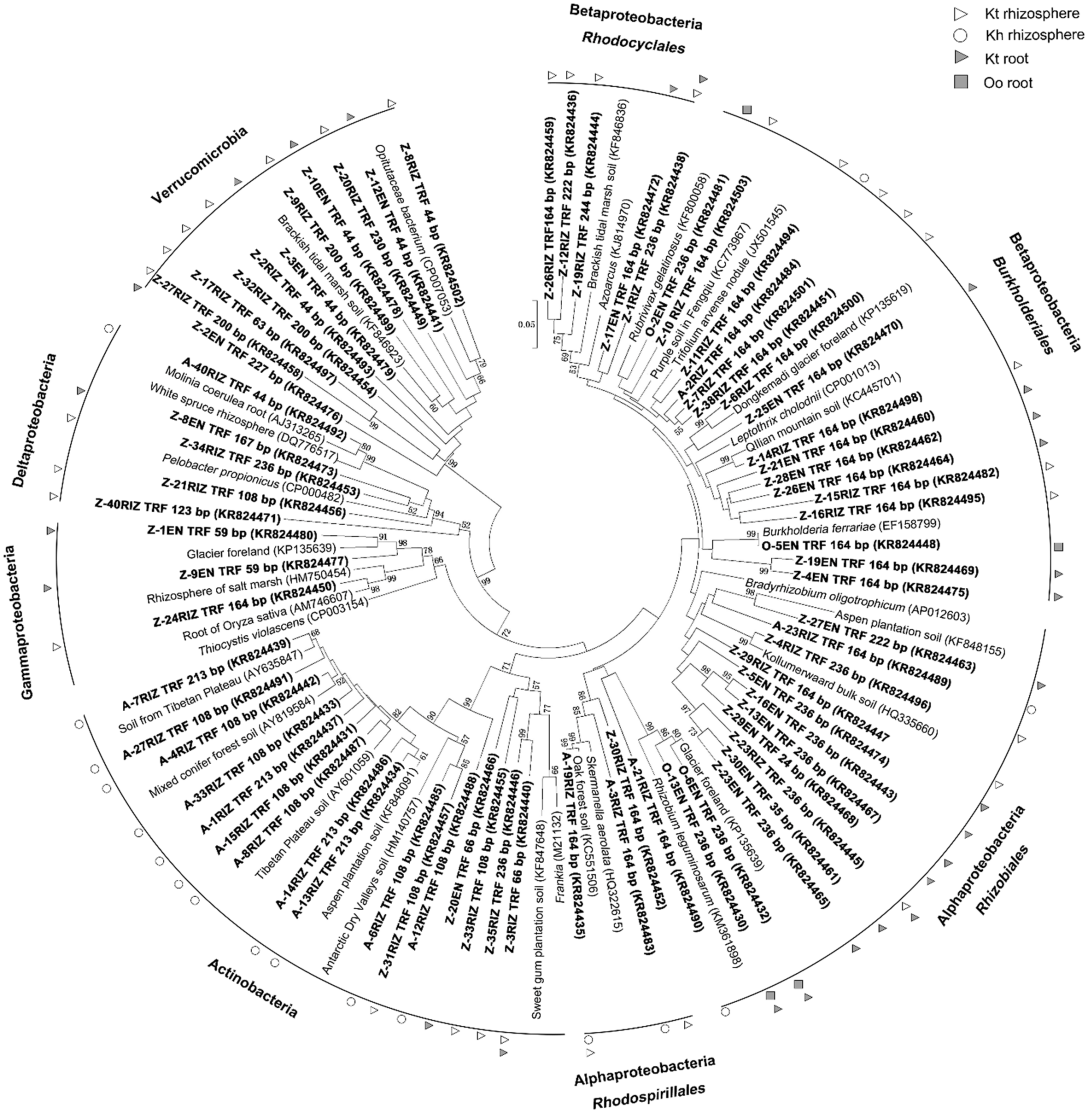
Neighbor-joining phylogenetic tree of *nif*H gene sequences. Clones are shown in bold with sample name, followed by the corresponding length of terminal restriction fragments and accession number. Bootstrap values (>50) determined from 1,000 iterations are indicated at branch points.

A total of 140 *nif*H gene sequences were retrieved, and 73 OTUs were detected and fell into 6 clusters. Alphaproteobacteria were the most abundant diazotrophs in the roots of *O. ochrocephala* and *K. tibetica*. Betaproteobacteria and Actinobacteria were significantly detected in the rhizosphere soils of *K. tibetica* and *K. humilis*, respectively. In addition, OTUs belonging to the phylum Verrumicrobia were abundantly observed in the rhizosphere soils and roots of *K. tibetica*. Furthermore, Gamma- and Delta-proteobacteria were mainly observed in the roots and rhizosphere soils of *K. tibetica* (Figure 3).

*Skermanella aerolata* and *Rhizobium leguminosarum* mainly dominated Alphaproteobacteria, whereas Burkholderiales and Rhodocyclales were the most abundant members of Betaproteobacteria. Furthermore, Actinobacteria diazotrophs were closely related to *Frankia,* which were abundantly observed in the rhizosphere soils. Diazotrophs related to *Thiocystis violascens*, *Pelobacter propionicus*, and Opitutaceae bacterial clusters dominated Gammaproteobacteria, Deltaproteobacteria, and Verrucomicrobia, respectively.

**Figure 4:**
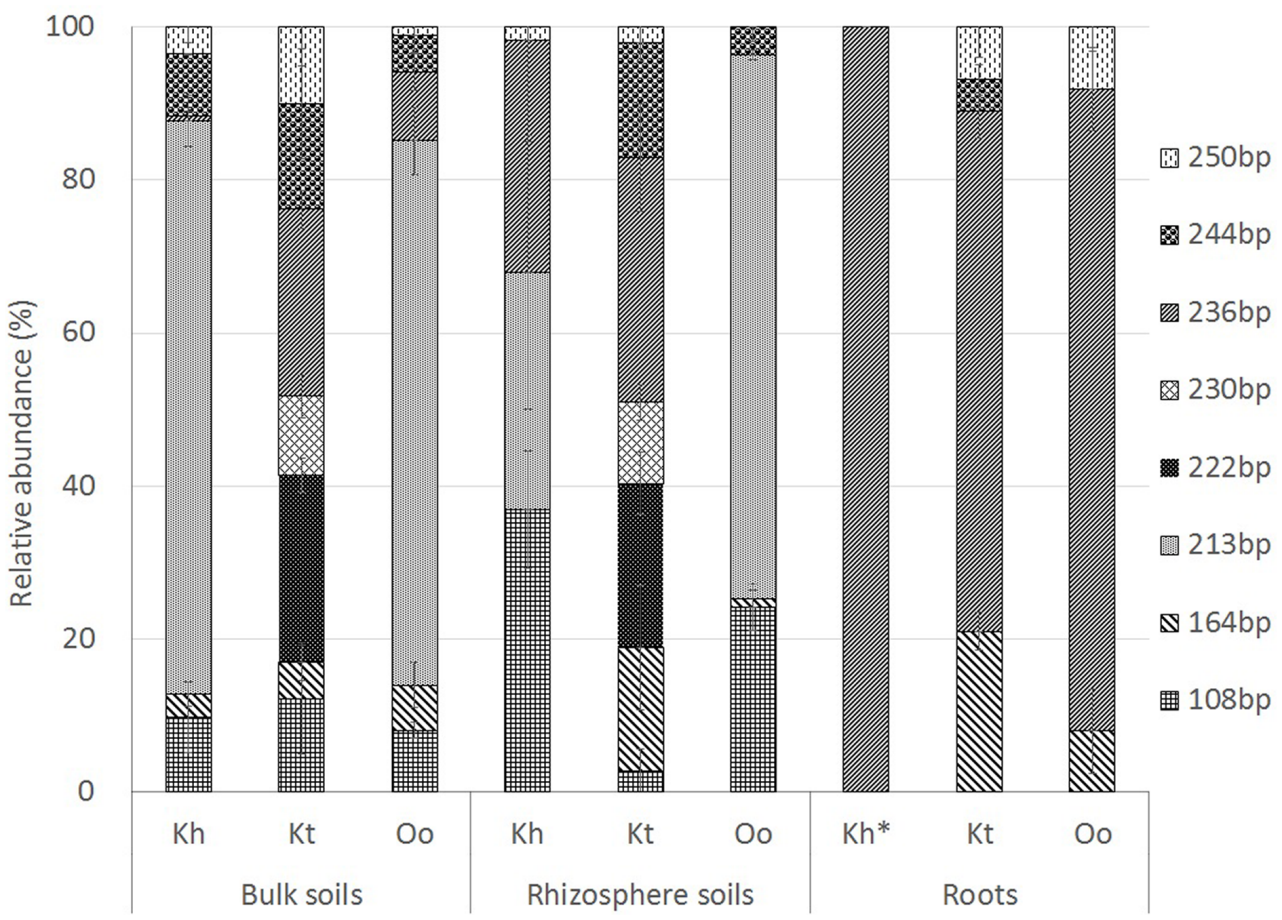
Relative abundance and distribution of the most dominant diazotrophic terminal restriction fragments obtained by terminal restriction fragment length polymorphism analysis. Kh, Oo and Kt represent *Kobresia humilis*, *Oxytropis ochrocephala*, and *Kobresia tibetica*, respectively.

In bulk and rhizosphere soils of *O. ochrocephala*, TRF 213 bp (~70%), assigned to Actinobacteria, was the dominant TRF. However, in *K. tibetica* bulk and rhizosphere soils, 6 TRFs identified in our study had a relatively high abundance (>10%), and TRFs 222 bp and 236 bp (~25%) were the major TRFs. These fragments were assigned to Rhizobiales (Alphaproteobacteria) and Rhodocyclales (Betaproteobacteria). Conversely, the community structures of *O. ochrocephala* and *K. tibetica* were dominated by TRF 236 bp, which occupied 84 and 68% of the total TRFs in the roots of *O. ochrocephala* and *K. tibetica*, respectively (Figure 4). In *K. tibetica* roots, the TRF 164 bp assigned to Betaproteobacteria (Burkholderiales) also occupied a large proportion (~20%). The Shannon diversity index was also used to differentiate the diazotrophic community structure. Bulk and rhizosphere soils had a more diverse diazotrophic community than the roots of three plants. *K. tibetica* was the most diverse among all samples, while *K. humilis* was the least diverse (Table 1).

**Table 1:** Diazotrophic diversity in bulk and rhizosphere soils and roots of *Kobresia humilis*, *Kobresia tibetica*, and *Oxytropis ochrocephala* based on terminal restriction fragment length polymorphism profiles.

	*Kobresia humilis*	*Kobresia tibetica*	*Oxytropis ochrocephala*
Bulk soils	0.91	1.83	1.03
Rhizosphere soils	1.22	1.69	0.76
Roots	0.42	0.91	0.55

The diazotrophic diversity of both bulk and rhizosphere soils was higher than that in the roots of all three plant species.

### Identification of highly efficient diazotrophs

To explore the probability of plant-diazotrophs in non-legume *K. tibetica* roots, the relative abundance of efficient and highly diazotrophs in each clone library was calculated. In the current study, the diazotrophs were classified as efficient or highly efficient diazotrophs based on previous publications (Noe and Hammerstein, 1994). Rhizobiales and Rhodocyclales were regarded as highly efficient diazotrophs for their potentials to form nodulation with plants, while Alphaproteobacteria (except Rhizobiales), Betaproteobacteria (except Rhodocyclales), and Actinobacteria were regarded as efficient diazotrophs (Gyaneshwar et al., 2011; Lu et al., 2012; Fernandez et al., 2014; Shu et al., 2015; Huang et al., 2021). In *O. ochrocephala* roots, all of the diazotrophs were efficient, and the majority (>90%) were identified as being highly efficient. A higher proportion of efficient (81%) and highly efficient (44%) diazotrophs was detected in *K. tibetica* roots compared with the rhizosphere soils, where 28% of the diazotrophs were identified as highly efficient. Although the majority of the diazotrophs were identified as efficient, only 5% of the total potential diazotrophs were identified as highly efficient in the *K. humilis* rhizosphere soils (Table 2).

**Table 2:** Relative abundance of diazotrophs based on clone library.

Diazotrophs	Rhizosphere soils		Roots	
	Kh	Kt	Kt	Oo
Efficient	97.44%	69.44%	81.48%	100.00%
Highly efficient	5.26%	40.00%	54.55%	93.10%

Alphaproteobacteria (except Rhizobiales), Betaproteobacteria (except Rhodocyclales) and Actinobacteria clusters are regarded as efficient diazotrophs, and Rhizobiales (Alphaproteobacteria) and Rhodocyclales (Betaproteobacteria) are regarded as highly efficient diazotrophs because they form strong symbiosis with specific legume and non-legumes (Gyaneshwar et al., 2011; Ribeiro et al., 2011; Lu et al., 2012; Fernandez et al., 2014). Kh, Oo, and Kt represent *Kobresia humilis*, *Oxytyopis ochrocephala*, and *Kobresia tibetica*, respectively.

## Discussion

In the current study, abundant diazotrophs were detected in bulk and rhizosphere soils and roots of the three plant species on the Qinghai-Tibet plateau. In both bulk and rhizosphere soils, *K. tibetica* showed the highest diazotrophic abundance, whereas *K. humilis* showed the lowest. The diazotrophic abundance of *K. tibetica* showed a similar level to that reported by Tai et al. (2013) in both rhizosphere and bulk soils, but substantially higher than Antarctic dry valleys soils (Niederberger et al., 2012). The legume plant *O. ochrocephala* showed substantially higher diazotrophic abundance within roots than *K. tibetica* and *K. humilis*, and the former was even higher than arable plant roots (Juraeva et al., 2006). The high diazotrophic abundance in soils and roots may suggest that these diazotrophs could potentially fix substantial nitrogen into soils in the plateau grassland, although nitrogen-fixing rate was not measured in the current study.

The *nifH* gene abundance detected in the bulk and rhizosphere soils was similar to that of the Arctic biological soil crust (BSC, 10^7^–10^8^ g^−1^ soil). The Arctic BSC had a high nitrogen fixation rate during the growing season (8.7–134 nmol C_2_H_4_ g^−1^ h^−1^) (Stewart et al., 2011). This contrasts with the findings of Keuter et al. (2014) and Li et al. (2019), which showed lower levels of nitrogen fixation rates in grassland soils, respectively. Diazotrophic abundance was significantly influenced by soil pH (Tai et al., 2013) and soil C: N ratio (Singh et al., 2011), and a strong correlation between soil moisture and the diazotrophic abundance was also observed (Che et al., 2018). Therefore, the high diazotrophic abundance could be attributed to the optimal environmental factors in bulk and rhizosphere soils on the plateau.

Diazotrophs were abundant in non-leguminous plant roots of *K. tibetica*. Previous studies have also reported abundant diazotrophs in non-legume plants. For example, *Oryza sativa L.* can form associations with various endophytic bacteria from the genus *Azoarcus* (Fernandez et al., 2014) and *Rhizobium leguminosarum* (Yanni et al., 1997). *Citrobacter amalonaticus* and *Xanthomonas translucens* have been isolated in the rhizosphere or tissues of the wild rice, *Oryza rufipogon.* Moreover, *Gluconacetobacter diazotrophicus* is known to fix nitrogen in sugarcane (Dobereiner, 1992) and *Ananas comosus* (Tapia-Hernandez et al., 2000). Therefore, these findings suggest that free-living diazotrophs in non-legume plants may play an important role in fixing nitrogen in the Qinghai-Tibet Plateau grasslands.

Herein diazotrophs were abundant in both the non-legume (*Kobresia tibetica* and *Kobresia humilis*) and legume (*Oxytropis ochrocephala*) plants. Phylogenetic analysis revealed that Proteobacteria (alpha-, beta-, gamma-, and delta-), Actinobacteria, and Verrumicrobia dominated the diazotrophic community. This is consistent with previous findings on the Qinghai-Tibet plateau that Proteobacteria were the key diazotrophs (Che et al., 2018). Similarly, Proteobacteria and Actinobaceria were the most abundant diazotrophs in switchgrass (Bahulikar et al., 2014), rhizosphere soil and the roots of *Lasiurus sindicus* grass in (Chowdhury et al., 2009). Diazotrophs have widely been observed in non-legume plants, including maize (Bloch et al., 2020; Wang et al., 2020), rice (Wang et al., 2020; Mir et al., 2022), wheat (Kariman et al., 2022) and sorghum (Barros et al., 2020), grasses (Pankievicz al., 2015) and sugarcane (Matoso et al., 2020). Furthermore, Proteobacteria were dominant diazotrophs in non-legume plant species, such as *Panicum coloratum*, *Chloris gayana* and *Digitaria eriantha* (Gupta et al., 2019). Our results showed that diazotrophs in *K. tibetica* were related to *Rhizobium leguminosarum* and A*zoarcus*, 44% of which were highly efficient diazotrophs. *K. tibetica* could thus form symbiotic associations with more diazotrophs than the leguminous *O. ochrocephala*. Consequently, this is agreement with previous studies that *K. tibetica* could gain extra nitrogen from these symbiotic relationships (Wang et al., 2012, 2014). Therefore, these findings indicate that these free-living diazotrophs occur widely in non-legume plants and soils and may potentially fix substantial nitrogen on the Qinghai-Tibet Plateau.

In our study, the roots of three different plants had similar diazotrophic community structure, consistent with previous studies, which showed similar diazotrophs in different plants. For example, sugarcane (*Saccharum* spp.) and rice (*Oryza sativa*) exhibited similar diazotrophic community structures in stem and roots (Thaweenut et al., 2011). Additionally, the diazotroph community structure and abundance showed little difference in diazotrophic community structures in wheat and pea (Reardon et al., 2014).

Exploring the abundance and community structure of diazotrophs is crucial to understanding the mechanisms that controls nitrogen levels in grassland ecosystems (Yang et al., 2011; Zhang et al., 2022). The abundant free-living diazotrophs in bulk and rhizosphere soils and roots suggest the essential roles of diazotrophs in fixing nitrogen into soils and plants in the Qinghai-Tibet Plateau grasslands. In particular, the diazotrophic associations may facilitate the growth and development of non-legume plants by providing additional nitrogen nutrition (Fowler et al., 2013). These associations could be important in nitrogen-deficient grassland ecosystems with less legume plants, because they can improve the productivity of non-legume plants (Carvalho et al., 2014). In the current study, we concentrated on the diazotrophic abundance and community structure, and did not measure nitrogen fixation rates, which are crucial for understanding the functions of free-living diazotrophs. Therefore, nitrogen fixation rates need to be considered in future studies (Sarkar and Reinhold-Hurek, 2014).

Understanding diazotrophic associations is important for biodiversity conservation and ecosystem management in grasslands with less legume plants. This is particularly important for the Qing-Tibetan Plateau, because the plateau grasslands are vulnerable (Wang et al., 2011; Zhao et al., 2011) and have been significantly degraded by climate change and human activities (Harris, 2010; Fassnacht et al., 2015; Zhang, 2018). The diazotroph associations facilitate the growth of specific plant species and could potentially accelerate the recovery of degraded grasslands by improving the ecosystem nitrogen nutrition. Therefore, the strategies of biodiversity conservation and ecosystem management can be adjusted by incorporating the diazotrophic associations with non-legume plants in the plateau grasslands.

This study used T-RFLP and clone library analysis for determining diazotrophic community structure and compositions. While T-RFLP method has widely been applied to explore diazotrophic and other microbial communities in diverse ecosystems for the low cost (Xiao et al., 2010; Li et al., 2016), although this method is constrained by PCR artifacts (Hewson and Fuhrman, 2006). High-throughput sequencing method provide higher resolution than TRFLP for microbial community, and has emerged as the most reliable approach to investigate microbial community structure (Song et al., 2022; Hao et al., 2023). High-throughput sequencing methods should be used in future studies.

## Conclusion

This study reported the diversity and abundance of free-living diazotrophs in Qinghai-Tibet grasslands. However, abundance, diversity, composition, and community structure were highly dependent on plant and soil type. The study provides insights into the role of free-living diazotrophs in biological nitrogen fixation, which is the major source of fixed nitrogen on the Qinghai-Tibet Plateau. The results of this study have shed light on why the soils of the Qinghai-Tibet grassland ecosystem are nitrogen-rich despite being dominated by non-leguminous plants. However, the study could only cover part of the area of the Qinghai-Tibet grassland ecosystem. Therefore, future studies should explore other remaining parts of this region.

## Data availability statement

The datasets presented in this study can be found in online repositories. The names of the repository/repositories and accession number(s) can be found at: https://www.ncbi.nlm.nih.gov/genbank/, KR824430 to KR824503.

## Author contributions

JN: Writing – original draft, Writing – review & editing, Data curation, Methodology. KZ: Data curation, Writing – original draft. WW: Data curation, Conceptualization, Funding acquisition, Methodology, Writing – review & editing. WK: Funding acquisition, Conceptualization, Writing – original draft, Writing – review & editing.

## Footnotes

^1^ www.ncbi.nlm.nih.gov/BLAST/
